# PI3Kδ Inhibition Enhances the Antitumor Fitness of Adoptively Transferred CD8^+^ T Cells

**DOI:** 10.3389/fimmu.2017.01221

**Published:** 2017-09-29

**Authors:** Jacob S. Bowers, Kinga Majchrzak, Michelle H. Nelson, Bulent Arman Aksoy, Megan M. Wyatt, Aubrey S. Smith, Stefanie R. Bailey, Lillian R. Neal, Jeffrey E. Hammerbacher, Chrystal M. Paulos

**Affiliations:** ^1^Department of Microbiology and Immunology, Medical University of South Carolina, Charleston, SC, United States; ^2^Department of Dermatology, Medical University of South Carolina, Charleston, SC, United States; ^3^Department of Surgery, Medical University of South Carolina, Charleston, SC, United States; ^4^Faculty of Veterinary Medicine, Department of Physiological Sciences, Warsaw University of Life Sciences, Warsaw, Poland; ^5^Department of Genetics and Genomic Sciences, Icahn School of Medicine at Mt Sinai, New York City, NY, United States

**Keywords:** adoptive cell therapy, T cell, cancer, memory, phosphatidylinositol-3-kinase, CAL-101, idelalisib

## Abstract

Phosphatidylinositol-3-kinase p110δ (PI3Kδ) inhibition by Idelalisib (CAL-101) in hematological malignancies directly induces apoptosis in cancer cells and disrupts immunological tolerance by depleting regulatory T cells. Yet, little is known about the direct impact of PI3Kδ blockade on effector T cells from CAL-101 therapy. Herein, we demonstrate a direct effect of p110δ inactivation *via* CAL-101 on murine and human CD8^+^ T cells that promotes a strong undifferentiated phenotype (elevated CD62L/CCR7, CD127, and Tcf7). These CAL-101 T cells also persisted longer after transfer into tumor bearing mice in both the murine syngeneic and human xenograft mouse models. The less differentiated phenotype and improved engraftment of CAL-101 T cells resulted in stronger antitumor immunity compared to traditionally expanded CD8^+^ T cells in both tumor models. Thus, this report describes a novel direct enhancement of CD8^+^ T cells by a p110δ inhibitor that leads to markedly improved tumor regression. This finding has significant implications to improve outcomes from next generation cancer immunotherapies.

## Introduction

Adoptive T cell transfer (ACT) therapy for cancer enriches and expands autologous tumor-reactive T cells before returning them to the patient. Thus, ACT allows for the *in vitro* selection or generation of T cells optimally suited to exert antitumor immunity *in vivo*, such as memory T cells. Less differentiated memory T cells (those that express high lymphoid homing molecules CD62L and CCR7), mediate robust antitumor responses resulting in better patient outcomes (1, 2). Conversely, not only are fully differentiated effector CD8^+^ T cells ineffective at clearing tumors (3) but also these effector cells will corrupt the antitumor potential of less differentiated T cells if cultured *ex vivo* together (4). The improved antitumor efficacy of less differentiated T cells is due in part to an improved capacity to engraft and persist long-term in the host. This T cell longevity may be due to not only improved trafficking to lymphoid tissues but also improved capacity to respond to homeostatic cytokines (5). Additionally, stem memory T cells (Tscm), which are the least differentiated of the memory subsets and express active Wnt/β-catenin signaling, possess the greatest capacity to clear tumors and provide long-term immunity (6).

A major pursuit in the cellular therapy field is to preferentially expand large numbers of T cells possessing a less differentiated state. Particularly exciting are methods that use small molecules that pharmaceutically enhance T cell memory during *ex vivo* expansion. Multiple emerging strategies include blockade of β-catenin degradation (6), increasing/strengthening mitochondrial networks to mimic those seen in memory T cells (7), denial of glucose *via* a small molecule inhibitor (8), and inhibition of the PI3K/AKT axis (9, 10).

The PI3K/AKT axis plays an integral role in T cell activation downstream of the TCR and costimulatory molecules. This pathway is important for T cell clonal expansion, survival, and cytokine production (11). The PI3K/Akt axis is also involved in memory formation, as AKT phosphorylates and sequesters FOXO transcription factors preventing transcription of CD62L, CCR7, CD127, and other molecules associated with less differentiated T cells (12). CD8^+^ T cells from patients who have an overactive p110δ rapidly proliferate and become terminally differentiated, leading to chronic inflammation and greater susceptibility to viral infection (13, 14). Yet this pathway is important for long-term memory formation of T cells, as T cells from mice with an inactive mutant p110δ subunit proliferate poorly and are less functional (15, 16). In particular, the few surviving memory cells in these mice are insufficient to mount a successful response to reinfection (17, 18).

Even though the PI3K/AKT pathway plays such a central role in effector T cell biology, the impact of p110δ inhibitors on cancer immunity has historically been associated solely with the promotion of effector CD8^+^ T cells due to ablating regulatory T cells (Tregs) after systemic drug administration (19). Yet, recent studies clearly show that adoptively transferred T cells treated *ex vivo* with a small molecule inhibitor of AKT, AKT inhibitor VIII (AKTi), exert stronger antitumor responses in both GVL and melanoma (9, 10). Moreover, we reported that *ex vivo* PI3Kδ inhibition with CAL-101 in a Th17 culture manifests a precursor lymphocyte population with a central memory-like phenotype and profoundly enhanced antitumor activity (20). Thus, it is possible that the therapeutic outcome of systemic CAL-101 therapy in cancer may be due to the direct enhancement of effector CD8^+^ T cell function in addition to selective Treg depletion. Indeed, both the genetic and pharmaceutical studies indicate a direct role of PI3K blockade on effector CD8^+^ T cells memory. However, it should be noted that while genetic perturbations seem to reduce effector memory capacity, pharmaceutical inhibition appears to paradoxically enhance memory phenotype. Additionally, though PI3K and AKT inhibition are often considered identical because of their linear signaling relationship, PI3K inhibition may change effector T cell physiology differently than AKT inhibition. For example, PI3K interacts with multiple other kinases besides AKT including MAPK and PKC kinases (21–23), as well as other AGC family kinases through the downstream kinase PDK1 (24). We therefore posited that pharmaceutical inhibition of p110δ might directly endow tumor-reactive CD8^+^ T cells with more durable memory properties than direct AKT inhibition.

We tested our hypothesis in both murine transgenic TCR pmel-1 CD8^+^ T cells and human peripheral blood T cells engineered with a tumor antigen specific chimeric antigen receptor (CAR). These cells were expanded with either the P110δ inhibitor CAL-101 or AKTi before adoptive transfer. We found that CAL-101 expanded central memory T cells (both murine and human) expressed even greater levels of CCR7, CD62L, and the alpha receptor for IL-7 (CD127) than AKTi. CAL-101-treated T cells had the highest levels of donor cells post-transfer, which corresponded with improved tumor control and lengthened survival. While CAL-101 treatment enriched T cells with high lymphoid homing receptors and responsiveness to IL-7, this could not explain the improved antitumor efficacy. CD62L^+^ T cells sorted from IL-2 expanded T cells could not recapitulate the potency of CAL-101 priming. Additionally, depletion of endogenous IL-7 in the host did not abrogate the capacity of CAL-101-primed T cells to clear tumor. However, deep sequencing of mRNA indicated that PI3Kδ blockade uniquely upregulated the stem memory transcription factor Tcf7 much more than AKT inhibition. These results imply that the simple application of CAL-101 in the preparation of effector T cells directly induces a memory program that greatly improves their capacity to control malignancies.

## Materials and Methods

### Mice and Cell Lines

C57BL/6J (B6), pmel-1 TCR transgenic mice, and NOD/scid/gamma chain knock out (NSG) mice were purchased from Jackson Laboratories and housed and bred in the Medical University of South Carolina Hollings Cancer Center (MUSC, Charleston, SC, USA) comparative medicine department. NSG mice were housed under specific pathogen-free conditions in microisolator cages and given autoclaved food and acidified water. Housing and experiments were conducted in accordance with MUSC’s Institutional Animal Care and Use Committee’s (IACUC) procedures. B16F10 (H-2b) melanoma tumor, gift of the Nicholas Restifo lab at NCI surgery branch was maintained in culture media (RPMI 1640 w/l-glutamine, 10% FBS, 1% Pen/strep, NEAA, and Na pyruvate, and 0.1% BME and Hepes). M108 xenograft tumors were a gift from the June lab at the University of Pennsylvania. M108 were cultured and engrafted as described previously (25).

### T Cell Cultures

*Pmel-1* CD8^+^ T cells were prepared from whole splenocytes activated using 1 µM hgp100 peptide + 100 IU rhIL-2/mL. Starting 3 h after initial activation, cells were treated with DMSO vehicle, AKT inhibitor VIII (Calbiochem), or CAL-101 (Selleckchem) at indicated doses. Cells were supplemented with culture media containing 100 IU rhIL-2/mL and vehicle or drug when expanded. *Human normal donor peripheral T cells*: polyclonal CD3^+^ T cells were prepared *via* negative bead selection (Dynal) from peripheral blood lymphocytes and activated using CD3/CD28 beads (Gibco) with 100 IU rhIL-2/mL DMSO vehicle, 1 or 10 μM AKTi or CAL-101 throughout culture as indicated. On day 2 of culture, T cells were engineered to be mesothelin specific *via* lentiviral transduction with an antimesothelin CAR which contained a single-chain variable fragment (scFv) fusion protein specific for mesothelin and linked to the T cell receptor ζ (TCRζ) signaling domain and 4-1BB as described previously (25) (gifts from the June lab). Tumor infiltrating lymphocytes (TILS): TILs were derived from non-small cell lung cancer tumor samples from two patient donors provided by Dr. John Wrangle and Dr. Mark Rubinstein. The tumors were rinsed in CM and cut into 1–3 mm pieces, which were individually transferred to wells of a 24-well plate containing 2 ml of TIL media (CM with a final concentration of 6,000 IU/mL recombinant IL-2). Each well was considered an individual TIL product for analysis. After 5–7 days, 1 ml of media was removed and replaced with 1 ml of fresh TIL media. TIL were monitored and either given fresh TIL media or split when confluent every 2–3 days for up to 5 weeks. On week 3, half of split wells were given CAL-101 (10 µM), while the original wells were treated with vehicle for the duration of the experiment.

### Adoptive Cell Therapy

*B6 mice* received 4.5e^5^ B16F10 cells subcutaneously 5–8 days before ACT. One day before therapy, mice underwent non-myeloablative 5 Gy total body irradiation. CD8^+^ T cells were *in vitro* activated with feeder cells and peptide 12 h before transfer as described (26) then infused *via* tail vein. Unless otherwise indicated, IL-2 complex was prepared at 1.5 μg rhIL-2 (NIH) and 7.5 μg anti-IL-2 antibody (clone JES6-1A12 BioXCell) per mouse and administered *via* intraperitoneal injections on days 0, 2, and 4 of treatment. Mice in the antibody neutralization experiment received 200 µg of either IL-7 neutralizing antibody (clone M25) or IgG2b isotype (clone MPC-11) (BioXCell) on days 0, 3, 5, 8, 12, and 17 of treatment *via* intraperitoneal injection as previously described (5). *NSG mice* received 6e^6^ M108 suspended in matrigel subcutaneously 51 days prior to adoptive therapy. On day of treatment, mice received 3.5–4e^5^ CAR T cells *via* tail vein injection. In all experiments, mice were randomized to treatment groups and tumor burden was monitored in blinded fashion using perpendicular caliper measurements. Tumor burden was reported as tumor area (mm^2^).

### Tissue Distribution Assays

Blood from treated mice was collected *via* cheek vein bleed then centrifuged to remove plasma and subjected to RBC lysis buffer (Biolegend), before being re-suspended in cell media for analysis. Spleens and draining (inguinal) lymph nodes were prepared *via* mechanical disruption followed by red blood cell lysis, and re-suspended for analysis. Tumors were sectioned, then mechanically disrupted and re-suspended in for analysis. Cell suspensions were blocked using FC block (Biolegend) at 1 μg/100 μL prior to probing with antibodies, then analyzed by flow cytometry.

### *In Vivo* Cytotoxicity Assay

C57BL/6 splenocytes were incubated with either hgp100 peptide or OTII peptide then labeled with a low level (0.5 μM) or high level (5 μM) of cell trace violet (Thermo Fisher Scientific), respectively. Hgp100 and OTII-pulsed splenocytes were mixed at equal ratio then injected intravenously into mice previously treated with T cells or untreated control mice. After 4 h, spleens were harvested from mice and the ability of donor cells to lyse hgp100-pulsed splenocytes vs. OTII-pulsed splenocytes was assessed *via* flow and reported as % specific lysis using the following equation: % specific lysis = [1 − (ratio of no T cell control mice)/(ratio of ACT mice)] × 100, where ratio = % OTII/% hgp100.

### Flow Cytometry and Cell Sorting

Flow cytometry was performed on a BD FACSverse instrument. Antibodies used for mouse cell analysis: CD127-PE/V450 clone A7R34, CD25-FITC clone 7D4, CD44-PerCPCy5.5/FITC clone IM7, CD69-PECy7 clone HI.2F3, CD8-PerCPCy5.5 clone 53-6.7, CD8-PECy7 clone YTS156.7.7, PD1-PerCPCy5.5 clone 29F.1A12 Vβ13-PE/APC clone MR12-3/MR12-4 (Biolegend), CD4-APCCy7 clone RM4-5, CD62L-APC clone MEL-14, and KLRG1-APC/V450 clone 2F1 (BD). Antibodies used for human cell analysis: CD127-PECy7 clone A019D5, CD25-APCCy7 clone BC96, CD4-APCCy7 OKT4, CD45RO-APC clone UCHL1, CD62L-FITC clone DREG-56, CD8-PerCPCy5.5 clone SK1, TIM3-PE clone F38-2E2 (biolegend), CCR7-PECy7 clone CCR7, CD8-V450 clone RPA-T8, and PD1-FITC clone M1H4 (BD Biosciences). Cell viability assessed *via* Zombie Live/Dead Stain (biolegend). Data analyzed using Flowjo 10 software (Tree Star). For experiments with sorted cells, CD8^+^Vb13^+^ T cells were sorted from pmel-1 cultures using Dynabeads untouched mouse CD8^+^ T cell kit (Invitrogen) and a FACSAria cell sorting machine (BD Biosciences).

### Western Blot

Nuclear protein from human normal donor CD3^+^ T cells cultured for 7–10 days was extracted *via* NE-PER nuclear and cytoplasmic extraction kit (Thermo Scientific) then subjected to Western blot. The following primary antibodies were used: mouse anti-h/m β-catenin clone 14/beta-catenin (BD), rabbit anti-h/m Histone H3 clone D1H2 (cell signal), rabbit anti-h/m Lef1 clone EP2030Y (abcam), and rabbit anti-h/m TCF7 clone EPR2035 (abcam). Quantification of optical density performed using Fiji analysis software (NIH).

### RNA Sequencing and Analysis

*Library preparation*: mRNA libraries were prepared in triplicate from each donor using the TruSeq RNA V2 kit (Illumina). Cleaved RNA fragments were copied into first strand cDNA then underwent second strand cDNA synthesis. End repair of cDNA fragments, single “A” base addition and ligation to the adapter followed. The product was then purified and enriched with PCR to create the final cDNA library. *Transcriptome sequencing*: cDNA libraries were clonally clustered onto the sequencing flow cell using the c-BOT (Illumina) Cluster Generation Station and Hiseq Rapid Paired-End Cluster Kit v2 (Illumina). Clustered flow cells were sequenced on the Illumina HiSeq2500 Sequencing System using the Hiseq Rapid SBS Kit V2 (Illumina). *Analysis*: Differential gene expression analysis contrasting the factors representing different treatment protocols (CAL101 vs. vehicle; or AKTi vs. vehicle) was performed by running kallisto on the raw paired-end RNA sequencing data (in FASTQ format) to estimate transcript-level read counts based on Ensembl GRCh37 cDNA assembly for each sample (NCBI GEO database Accession Number: GSE101497). Estimated transcript-level counts were aggregated across official gene symbols using the tximport package with mapping provided by the biomaRt utility. To better isolate the effects of drug treatments from the effects that can be attributed to differences in donors, we used both factors in our modeling of the gene-level count levels and used limma voom package to fit our model following their published standard protocol (27).

### Statistics

Kaplan–Meier survival curves were assessed for significance using a log rank test between treatment groups. A *p*-value of <0.05 was considered significant. Statistical comparisons between groups were performed *via* a Student’s *t*-test for two groups, or a one-way ANOVA followed by multiple comparisons of group means (3+ groups). A *p*-value of <0.05 was considered significant. All statistics reported as mean ± SEM. Statistical analysis of differential gene expression was performed using Bonferroni-adjusted *p*-values to account for multiple hypothesis testing and a cutoff at 0.05 for the adjusted values to assign significance of the differential expression across conditions.

### Study Approval

Studies were approved by the IACUC of the Medical University of South Carolina Animal Resource Center (ARC #3039). De-identified human PBMCs and tumor samples were collected under approval of the MUSC Internal Review Board (pro13570). Human T cells were engineered *via* approval from the Institutional Biosafety Committee (#2335).

## Results

### Pharmaceutical Inhibition of p110δ in pmel-1 CD8^+^ T Cells Increases Both CD62L and CD127 Expression without Hindering Expansion

**Figure 1:**
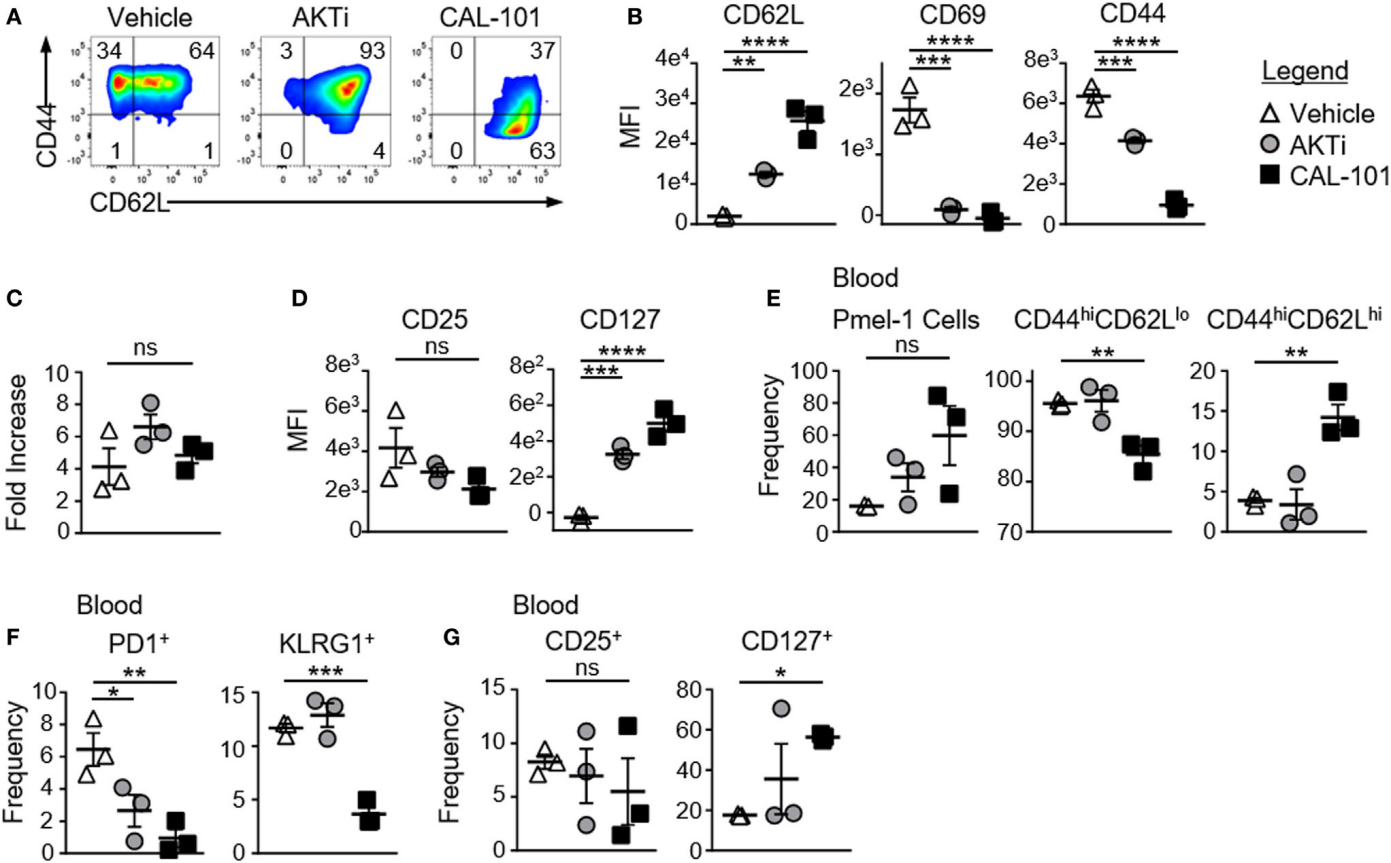
CAL-101 priming supports a less differentiated memory phenotype including high IL-7Ra expression on pmel-1 CD8^+^ T cells. **(A)** Representative flow plots of CD44 by CD62L expression on pmel-1 CD8^+^ T cells primed with vehicle, AKTi, or CAL-101 after 5 days of culture; representative of three independent cultures. **(B)** MFI of CD62L, CD69, and CD44 on pmel-1 CD8^+^ T cells after 5 days of culture; *n* = 3 independent cultures. **(C)** Fold increase of pmel-1 CD8^+^ T cells primed with vehicle (DMSO), AKTi, or CAL-101 5 days following antigen stimulation; *n* = 3 independent cultures. **(D)** MFI of CD25 and CD127 on pmel-1 CD8^+^ T cells after 5 days of culture; *n* = 3 independent cultures. **(E–G)** Frequency of donor pmel-1 T cells (infused on day 0 of treatment at 8 × 10^5^ cells/mouse) and extracellular markers on those cells in blood of tumor bearing B6 mice preconditioned with 5 Gy total body irradiation 7 days following treatment; *n* = 3 mice/group from one experiment. One-way repeated measures ANOVA; ns, not statistically significant, *p < 0.05, **p < 0.01, ***p < 0.001, and **** *p* < 0.0001.

To test whether PI3Kδ inhibition enhanced the antitumor capacity of T cells similarly to direct AKT inhibition, CD8^+^ T cells from pmel-1 transgenic mice (CD8^+^ T cells with a transgenic TCR specific for the melanoma/melanocyte antigen gp100) were activated with their cognate antigen and treated with CAL-101 throughout culture. As controls, T cells were expanded without drug (vehicle) or with AKT inhibitor VIII (AKT 1/2 hereafter denoted as AKTi), a published method to enhance T cell memory and antitumor efficacy (10). As expected, drug treatment induced marked differences in memory. While 65% of vehicle-treated CD8^+^ T cells expressed CD62L, nearly all cells treated with AKTi or with CAL-101 expressed CD62L (Figure 1A). However, CAL-101 exerted a stronger impact on the cells than direct AKT inhibition. The MFI of CD62L on CAL-101-treated T cells was higher than on those treated with AKTi. While both drugs decreased the activation marker CD69, only CAL-101 reduced CD44 expression (Figure 1B). While this CD44^lo^CD62L^hi^ phenotype mirrored the phenotype of a naive T cells, CAL-101-treated cells expanded similarly to both vehicle and AKTi-treated cells. These data suggest that despite PI3Kδ inhibition, T cell receptor and downstream proliferative signaling were intact in pmel-1 CD8^+^ T cells *in vitro* (Figure 1C).

### CAL-101-Treated CD8^+^ T Cells Robustly Engraft *In Vivo* and Maintain CD62L and CD127 Expression

While AKTi and CAL-101 slightly (but not significantly) reduced CD25 (IL-2 receptor alpha) on pmel-1 CD8^+^ T cells, these small molecules substantially elevated CD127, the alpha receptor for IL-7 (Figure 1D). Since IL-7 signaling is important for naive and central memory T cell homeostasis (28), and supports donor T cells in the host (5), we posited that the memory T cells generated from either CAL-101 or AKTi treatment would show superior engraftment and subsequent antitumor immunity compared to vehicle. Interestingly, CAL-101 pmel-1 CD8^+^ T cells engrafted with increased frequency in the blood compared to AKTi pmel-1 CD8^+^ T cells (Figure 1E). Additionally, while both AKTi and CAL-101 increased CD62L expression *in vitro* (Figure 1B), only CAL-101-treated pmel-1 retained significant levels of CD44^hi^CD62L^hi^ with fewer CD44^hi^CD62L^lo^ circulating cells (Figure 1E). Although both AKTi and CAL-101 T cells expressed less PD1 than vehicle *in vivo*, only CAL-101 T cells maintained reduced frequencies of the exhaustion marker KLRG1 (Figure 1F). Infused CD8^+^ T cells also retained far more CD127 on their cell surface *in vivo* when primed *ex vivo* with CAL-101 compared to untreated or AKTi-treated pmel-1 CD8^+^ T cells (Figure 1G). CAL-101 donor T cells were also detected at higher levels within the spleen and draining (inguinal) lymph nodes of tumor-bearing mice (not shown). Thus, we found that CAL-101-treated CD8^+^ T cells robustly engraft *in vivo*, maintain CD62L and CD127 expression and express lower levels of exhaustion markers.

### CAL-101 T Cells Impair Tumor Growth and Prolong Survival

**Figure 2:**
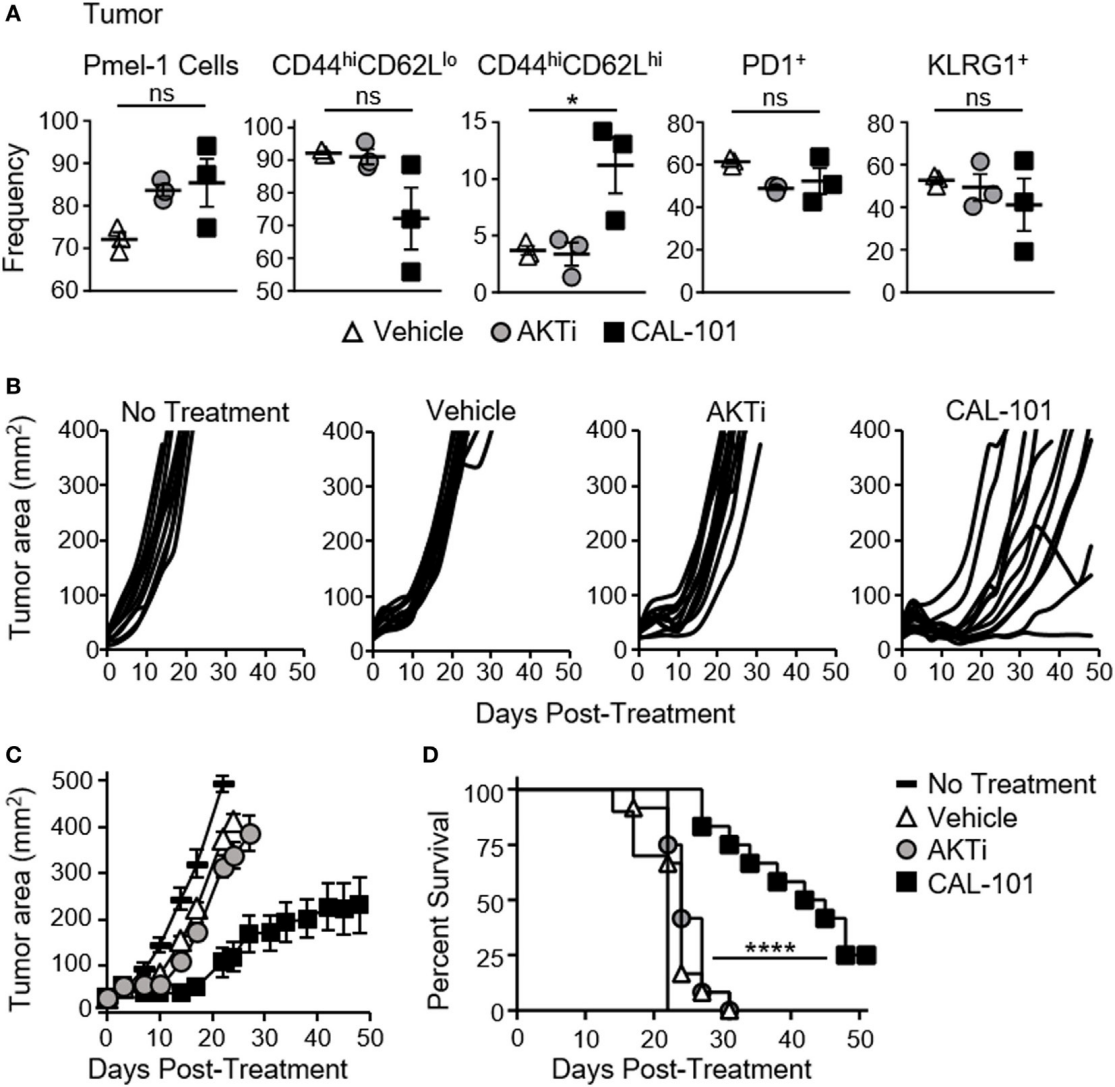
CAL-101-primed pmel-1 CD8^+^ T cells exert robust antitumor response against B16F10 tumors. **(A)** Frequency of donor pmel-1 T cells and extracellular markers on pmel-1 (vβ13^+^) donor T cells in tumor 7 days following treatment; *n* = 3 mice/group. One-way repeated measures ANOVA; ns, not statistically significant, *p < 0.05. Tumor burden (mm^2^) of **(B)** individual mice and **(C)** Average tumor burden (mm^2^) of treatment groups which received no T cell treatment, or 8 × 10^5^ pmel-1 CD8^+^ T cells primed with vehicle, AKTi, or CAL-101 *ex vivo*; *n* = 10–12 mice/group in one experiment. **(D)** Percent survival of above treatment groups. Kaplan–Meier curve analyzed by log rank test; ****p < 0.0001 (see also Figure S1 in Supplementary Material).

We suspected that the less differentiated memory phenotype of pmel-1 T cells fostered by CAL-101 treatment would translate to their improved ability to infiltrate and regress tumor once infused into melanoma-bearing mice. As expected, both CAL-101- and AKTi-treated T cells were detected at higher levels in the tumor compared to control (Figure 2A). More CAL-101 donor cells expressed a central memory phenotype within the tumor, however, the percentage of PD1^+^ and KLRG1^+^ donor cells were similar between groups (Figure 2A). Importantly, CAL-101-primed T cells were the most effective at slowing growth of melanoma in mice (Figures 2B,C), extending the lifespan of the animals beyond the survival of the vehicle or AKTi groups (Figure 2D). In fact, the tumor control exerted by AKTi-treated T cells was only slightly improved over tumor control by vehicle-treated T cells (Figure 2C). Since we were using a 10-fold higher drug dose in the CAL-101 cultures compared to the published amount of AKTi (10 vs. 1 μM) we posited that the difference in antitumor activity could be due to higher AKT inhibition by the elevated concentration of CAL-101. To test this idea, we treated pmel-1 CD8 T cells with either 1 or 10 μM of AKTi or CAL-101. Increasing the amount of AKTi to 10 μM marginally improved the antitumor efficacy of the T cells similar to treatment with 1 μM CAL-101 (Figure S1A in Supplementary Material). However, 10 µM CAL-101 treatment markedly improved tumor control and significantly improved survival compared to both 10 μM AKTi and 1 µM CAL-101 (Figures S1A,B in Supplementary Material). Additionally, while neither 10 µM AKTi nor 10 μM CAL-101 impaired the logarithmic expansion of mouse pmel-1 CD8^+^ T cells (not shown), we found that 10 μM AKTi dramatically inhibited growth of human T cells (Figure S1C in Supplementary Material). Thus, as high dose AKTi did not profoundly improve antitumor T cell potency against large melanoma tumors but did compromise the overall yield of human T cell cultures, we proceeded with comparisons of 1 μM AKTi to 10 μM CAL-101 in our remaining studies.

### CAL-101 Induces a Stronger Central Memory Phenotype than AKTi

**Figure 3:**
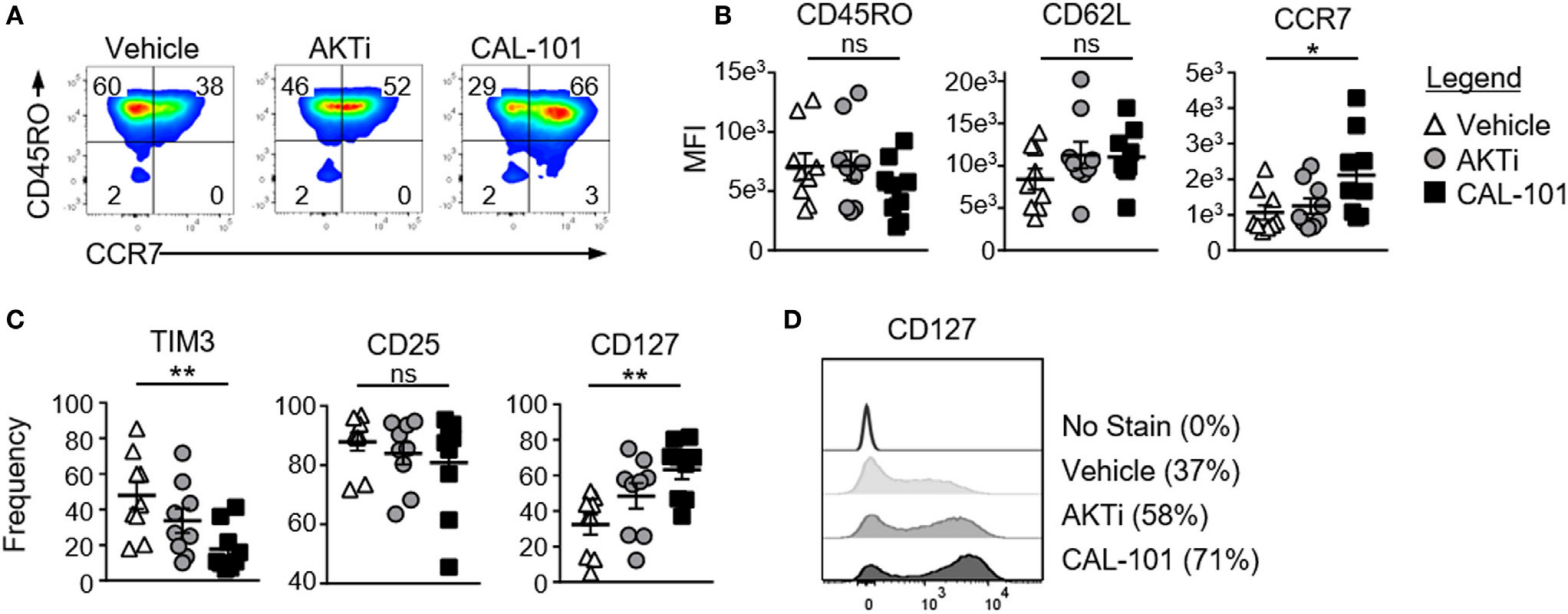
Phosphatidylinositol-3-kinase p110δ (PI3Kδ) and AKT blockade induce a central memory phenotype in human chimeric antigen receptor (CAR) CD3^+^ T cells. **(A)** CD44 and CD62L expression on vehicle, AKTi, or CAL-101-treated T cells from normal donor PBMC; representative of nine donors. **(B)** MFI of memory markers and **(C)** frequency of human CD3^+^ T cells expressing TIM3, CD25, and CD127; *n* = 9 normal donors. One-way repeated measures ANOVA; ns, not significant, *p < 0.05, **p < 0.01. **(D)** Histogram of CD127 expression on vehicle, AKTi, or CAL-101-treated T cells compared to no stain with frequency of positive cells indicated next to legend; representative of nine donors.

We next examined whether inhibiting PI3Kδ would augment the fitness and memory properties of human T cells. To do this, human CD3^+^ T cells were expanded with CD3/CD28 beads under CAL-101 or AKTi and compared to a vehicle control. All three T cell groups expressed similar CD45RO levels, a marker of T cell maturation. Both AKTi- and CAL-101-treated T cells had slightly increased CD62L over vehicle, though only CAL-101-primed T cells had significantly higher CCR7 (Figures 3A,B). PD1 was nominally expressed on all groups (not shown), but both drug treatments prevented the upregulation of coinhibitory receptor TIM3 post-activation compared to untreated T cells. Interestingly, CAL-101 significantly reduced TIM3 (Figure 3C). PI3Kδ and AKT blockade did not impact the expression of CD25, but only CAL-101 greatly increased CD127 on human T cells (Figures 3C,D). Collectively, our data reveal that CAL-101 supports the generation of human T cells with a central memory phenotype with reduced markers of inhibition compared to treatment with AKTi.

### PI3Kδ Blockade Augments the Antitumor Activity of Human CAR T Cells

**Figure 4:**
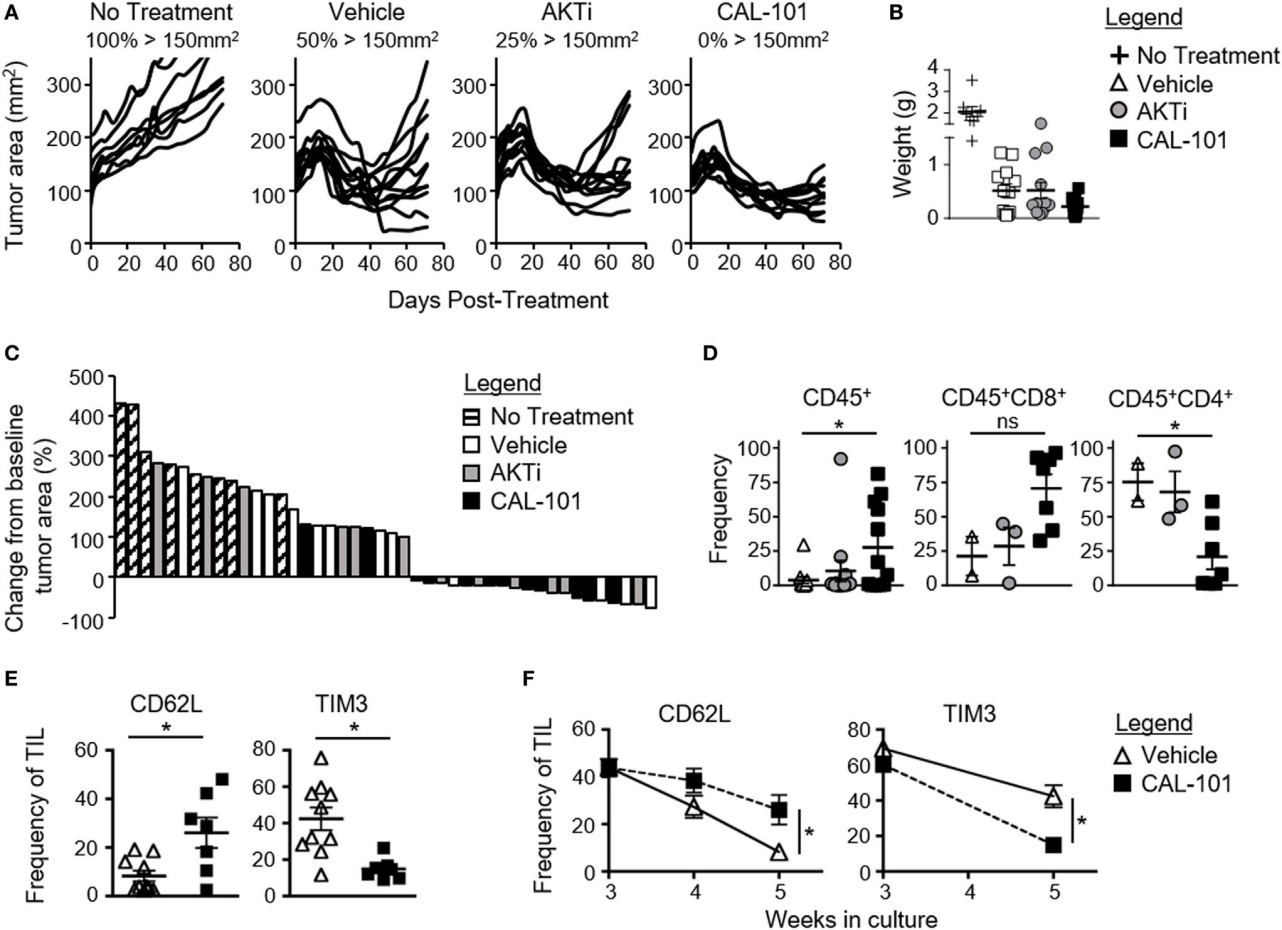
CAL-101 treatment improves tumor control by human chimeric antigen receptor (CAR) T cells compared to vehicle and AKTi treatment. **(A)** Individual tumor curves of NSG mice given M108 subcutaneously 51 days prior were treated with 4 × 10^5^ CD3^+^ mesoCAR T cells primed with vehicle, AKTi, or CAL-101; *n* = 8–12 mice/group in one experiment. **(B)** Tumor weight at day 71 posttransfer. **(C)** Percent change in size of tumors 71 days posttreatment compared to baseline tumor measurement at time of treatment. **(D)** Frequency of human CD45^+^ lymphocytes within the blood of treated mice 55 days posttransfer; *n* = 2–12 mice/group from one experiment. One-way repeated measures ANOVA; ns, not statistically significant, *p < 0.05. **(E)** Frequency of CD62L^+^ or TIM3^+^CD3^+^ tumor infiltrating lymphocyte (TIL) from lung carcinoma after 5 weeks of growth with either 2 weeks of CAL-101 treatment or not, and **(F)** change in frequencies of CD62L^+^ or TIM3^+^CD3^+^ TIL during drug treatment with CAL-101 or without (vehicle) from week 3 to week 5 of *ex vivo* culture. Groups compared by Student’s *t*-test, *p < 0.05.

We posited that human tumor-reactive T cells treated with CAL-101 *in vitro* would control the growth of human tumors in NSG mice better than donor vehicle or AKTi T cells. To address this idea, we transduced human T cells with a lentiviral vector containing a CAR that recognizes mesothelin plus 4-1BB and CD3ζ signaling domains (25). These CAR T cells were expanded for seven days with CD3/CD28 beads and IL-2 in the presence or absence of CAL-101 or AKTi before transfer into mice bearing subcutaneous M108 mesothelioma tumor. While all treatment groups initially reduced tumor burden in the mice, CAL-101-treated CAR^+^ T cells exerted longer tumor control compared to the vehicle- or AKTi-treated groups (Figure 4A). Conversely, half of tumors in vehicle T cell-treated mice, and a quarter of AKTi T cell-treated mice relapsed above 150 mm^2^ (Figure 4A). The superior antitumor immunity from CAL-101-primed T cells was further evidenced by the majority of tumors in CAL-101 mice having the smallest mean tumor weight (Figure 4B) and remaining below baseline measurement at the end of study (Figure 4C). Additionally, CAL-101 T cells persisted at significant levels in circulation 55 days after transfer in most treated mice (Figure 4D). This finding with CAL-101 was in stark contrast to vehicle and AKTi T cell-treated mice, which showed low levels of persisting cells in the mice (Figure 4D). Thus, priming T cells with CAL-101 improves engraftment, persistence, and tumor destruction by human CAR T cells in solid tumors.

Since PI3Kδ inhibition improved the antitumor efficacy of healthy donor-derived CAR T cells, we posited that CAL-101 treatment would also improve the memory phenotype of TILs from patients with lung carcinoma. To address this concept, individual TIL cultures were expanded under IL-2 for three weeks then split into CAL-101 or vehicle treatment groups for another 2 weeks. We found that CAL-101 supported the generation of TIL with higher CD62L but low TIM3 expression than vehicle after 5 weeks of expansion (Figure 4E). This phenotype appeared to be due to preservation of a less differentiated memory phenotype as vehicle TIL lost expression of CD62L faster than CAL-101 TIL (Figure 4F). Additionally, while TIM3 expression on both groups diminished during *ex vivo* expansion, CAL-101 treatment mediated a rapid loss of TIM3 on TILs (Figure 4F). Collectively, our data reveal that PI3Kδ blockade augments the antitumor activity of human CAR T cells and fosters the generation of TILs with a less exhausted and preserved memory profile.

### Antitumor Potency Induced by PI3Kδ Inhibition Is Not Due to CD62L

**Figure 5:**
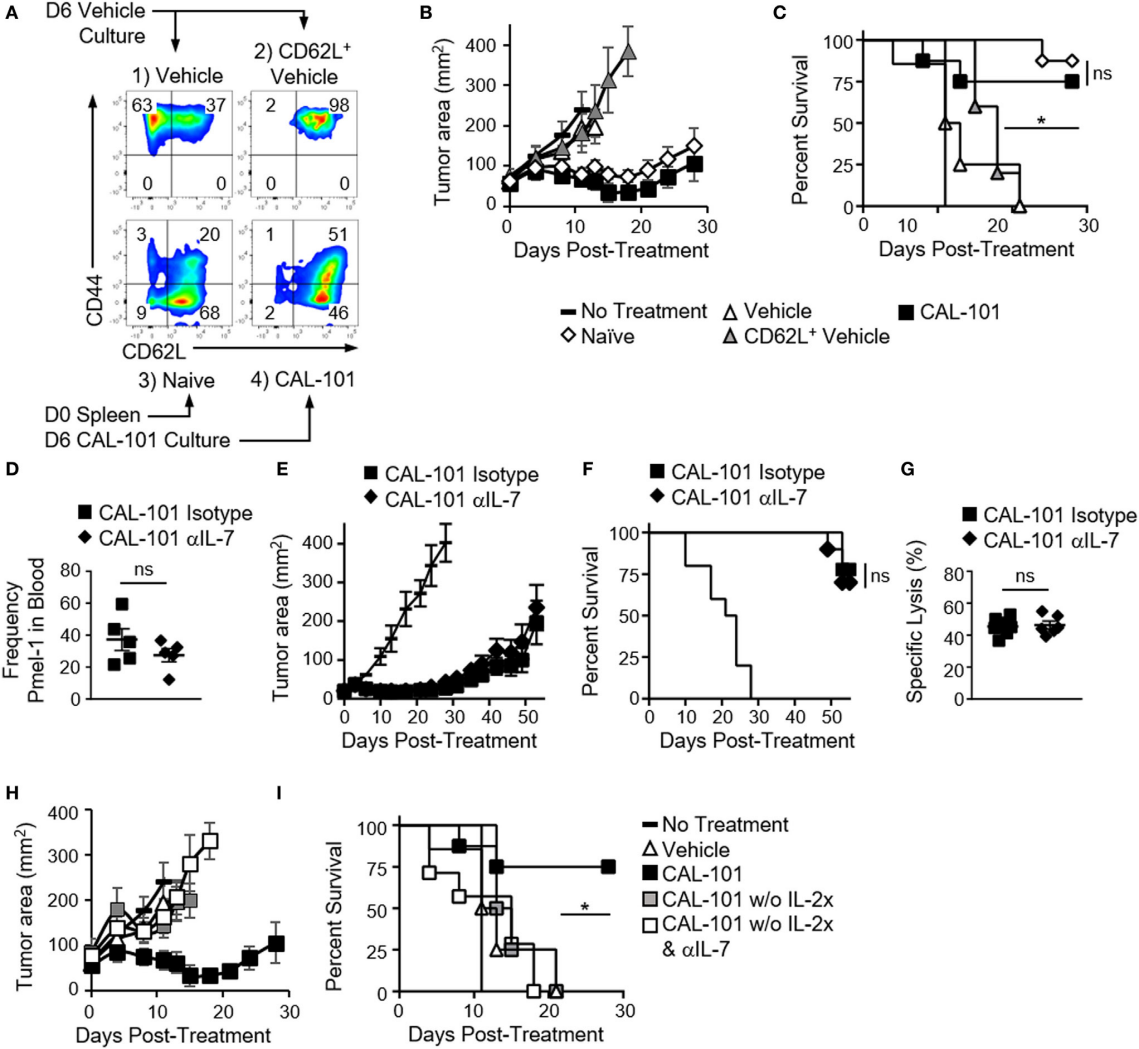
The antitumor potency of CAL-101-primed T cells is CD62L and CD127 independent but IL-2 dependent. **(A)** Sort diagram with postsort analysis of CD62L and CD44 expression on pmel-1 T cells. **(B)** Average tumor burden (mm^2^) and **(C)** percent survival of mice with B16F10 were treated with 1 × 10^6^ bulk vehicle, CD62L^+^ vehicle, naive, or CAL-101 pmel-1 cells compared to no treatment; *n* = 5–10 mice/group/experiment, representative of two independent experiments. Kaplan–Meier curve analyzed by log rank test; *p < 0.05. **(D)** Frequency of donor CAL-101 T cells in mice receiving isotype or IL-7 depleting antibody; *n* = 5 mice/group. One-way repeated measures ANOVA; ns, not statistically significant (see also Figure S2 in Supplementary Material). **(E)** Average tumor burden and **(F)** percent survival of isotype or IL-7-depleted mice receiving CAL-101-primed donor cells; *n* = 10 mice/group/experiment, representative of two independent experiments. Kaplan–Meier curve analyzed by log rank test; ns, not statistically significant. **(G)** Percent specific lysis of hgp100-loaded splenocytes by CAL-101-primed pmel-1 T cells in isotype-treated or IL-7-depleted mice 53 days posttransfer; *n* = 6–7 mice/group from one experiment. Comparison by Student’s *t*-test; ns, not statistically significant. **(H)** Average tumor burden and **(I)** percent survival of mice receiving CAL-101-treated donor cells with IL-2 complex, without IL-2 complex plus isotype, or without IL-2 complex plus IL-7 depletion compared to no treatment; *n* = 7–9 mice/group in one experiment. Kaplan–Meier curve analyzed by log rank test; *p < 0.05.

CD62L expression on T cells correlates with improved antitumor immunity in preclinical ACT tumor models (1, 3, 4, 29, 30). Moreover, enriching central memory T cells from peripheral blood and redirecting them with a CD19 specific CAR has shown efficacy in a clinical trial (2). Our studies corroborated these reports by showing a correlation between retained CD62L expression *in vivo* by CAL-101-treated donor cells and prolonged tumor control (Figures 1 and 2). Consequently, we naturally hypothesized that CAL-101-induced CD62L on T cells was responsible for their enhanced antitumor potency. Thus, if this concept were true, we suspect that simply sorting the CD62L^+^ T cells from vehicle pmel-1 should mirror the antitumor activity of CAL-101-primed T cells, which express high CD62L. To test this, the following pmel-1 T cell cohorts were administered to B16F10 mice: (1) bulk vehicle T cells (which were 37% CD62L^+^), (2) sorted CD62L^+^ T cells from vehicle-treated T cells (98% CD62L^+^), (3) naive pmel-1 T cells sorted directly from the spleen (majority CD44^−^CD62L^+^), and (4) CAL-101-treated T cells (which were 97% CD62L^+^, see Figure 5A).

In contrast to our hypothesis, we found that sorting CD62L^+^ pmel-1 cells from vehicle cultures did not improve treatment outcome over therapy with bulk vehicle. In contrast, CAL-101 mediated prolonged antitumor control (Figure 5B) and better survival (Figure 5C) in mice. Interestingly, naive sorted T cells closely matched the CAL-101-treated T cells in antitumor potency. It is important to note that despite similar antitumor efficacy, the benefit of CAL-101 expanded T cells over enriched naive T cells is the potential for propagating significantly more cells (i.e., higher cell yield) as vast numbers of cellular product are paramount for successful ACT therapy (3, 31). Nonetheless, these results imply that PI3Kδ blockade during *in vitro* proliferation might preserve naive T cell qualities, which would otherwise be corrupted by the expansion process. This finding also corroborates our data from TIL indicating a slower decline in CD62L in CAL-101-treated TIL. Thus, while T cells capable of long-lived memory responses against tumor express CD62L, enriching cells after *ex vivo* expansion that expresses this molecule is not sufficient to drive a successful antitumor response.

### CAL-101-Primed T Cells Regress Tumor Independent of IL-7 Signaling

Next, we tested whether the preserved CD127 on CAL-101-treated T cells was responsible for the robust antitumor properties *in vivo*. PI3Kδ blockade induced CD127 on murine and human T cells *in vitro*. Moreover, CD127 was sustained on CAL-101-treated T cells after transfer (Figures 1E and 2A). We posited that CAL-101 T cells thrived *in vivo* due to their enhanced responsiveness to IL-7. To test the dependence of CAL-101 antitumor responses on IL-7 signaling, we depleted IL-7 in the pmel-1 B16F10 model for the first two weeks after transfer as published (5). We suspected that depleting IL-7 would decrease engraftment of the donor cells and impair their control of tumor growth. In contrast, we found that while CAL-101-primed T cells engrafted at significantly higher numbers than vehicle or AKTi-treated T cells in both isotype and IL-7-depleted mice (Figure S2A in Supplementary Material), there was no significant difference between the CAL-101 treatment groups in frequency or memory phenotype (Figure 5D; Figures S2B,C in Supplementary Material). CAL-101-primed T cells also exerted their prolonged antitumor immunity whether IL-7 had been depleted or not (Figure 5E) resulting in no differences in survival between the CAL-101 T cell groups (Figure 5F). Furthermore, while IL-7 is important for maintaining naive and central memory T cell populations, we found no reduction in memory capacity of donor CAL-101 T cells in either isotype or IL-7-depleted animals, as both groups were equally capable of lysing tumor in mice (Figures 5E,F) and ablating hgp100 antigen bearing splenocytes in our very sensitive *in vivo* cytotoxicity assay (Figure 5G).

As IL-2 complex was administered to our melanoma tumor-bearing mice to support the infused CAL-101 T cells, we suspected that this cytokine was important for the engraftment of these infused cells and could compensate for IL-7. IL-2 complex has been reported to support the engraftment and proliferation of CD8^+^ T cells in ACT murine models (32). Thus, we posited that removal of IL-2 complex from the treatment protocol would reveal the importance of IL-7 signaling in the antitumor efficacy mediated by CAL-101 T cells. As expected, tumors grew more rapidly in mice that received CAL-101 T cells without IL-2 complex, compared to those which received both the pmel-1 CD8^+^ T cells and IL-2 complex (Figure 5H). However, because none of the pmel-1 T cell treatments without IL-2 complex were therapeutic, we were unable to truly assess whether IL-7 depletion compromised the antitumor activity of CAL-101 pmel-1 T cells in the absence of exogenous IL-2 administration (Figures 5H,I). Nonetheless, these experiments highlight the central importance of bolstering IL-2 signaling for effective antitumor function of adoptively transferred CD8^+^ T cells treated with CAL-101. Collectively, our findings lend further evidence that improved antitumor T cell efficacy from CAL-101 treatment is not likely due to their increased responsiveness to IL-7 signaling due to high CD127 expression nor was their improved potency due solely to their heighted expression of CD62L.

### CAL-101 Induces Stem Memory Pathways in T Cells while AKTi Does Not

**Figure 6:**
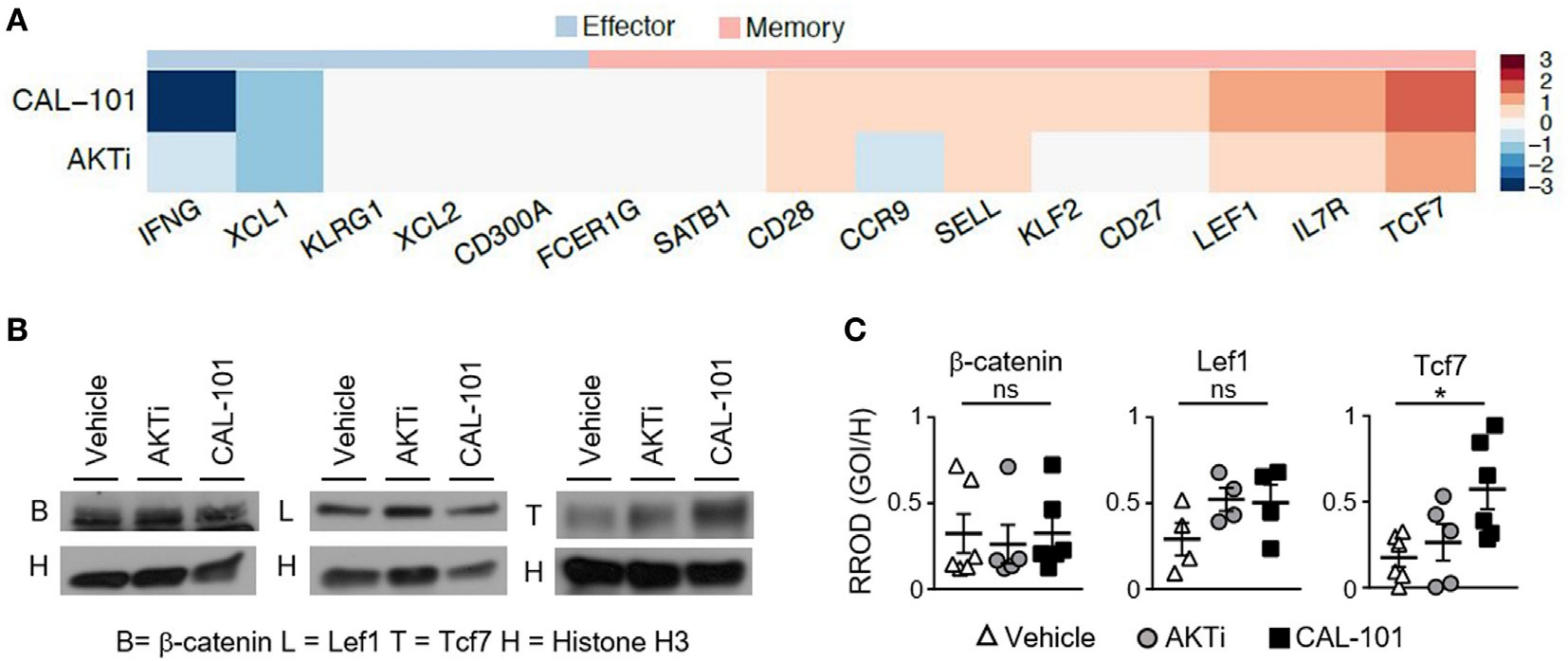
Human T cells primed with CAL-101 share transcriptional characteristics with stem memory lymphocytes. **(A)** Differential expression of memory and effector-associated genes in CAL-101 or AKTi-treated human T cells compared to vehicle analyzed using RNA sequencing (see also Figure S3 in Supplementary Material); *n* = 3 normal donors. **(B)** Western blot of nuclear protein extracts from vehicle, AKTi, or CAL-101-treated human T cells, B = β-catenin, L = Lef1, T = Tcf7, H = Histone H3; representative of 4–6 donors. **(C)** Quantified protein levels relative to Histone H3, RROD GOI/H = relative ratio of optical density (gene of interest over Histone H3); *n* = 4–6 normal donors. One-way repeated measures ANOVA; ns, not statistically significant, *p < 0.05.

To define how CAL-101 instills infused CD8^+^ T cells with enhanced antitumor activity *in vivo*, we next sought to use RNA sequencing to uncover the factors potentially responsible for the efficacy of this ACT therapy. We surveyed the differential expression of RNA transcripts of interest associated with memory and effector phenotypes, as well as other pathways influenced by drugs that block PI3K or AKT, including signaling intermediates, metabolic, anti-apoptotic pathways and cell cycle proteins. Similar to our protein data, we found that PI3Kδ inhibition *via* CAL-101 promoted the upregulation of multiple central memory markers on T cells, such as CD62L (*Sell*) and CCR7 compared to AKTi or vehicle-treated T cells. CAL-101 also uniquely induced high CD127 (*IL7r*) transcript and stem memory-associated transcripts Lef1 and Tcf7, which were markedly increased compared to AKTi cells (Figure 6A; Figure S3A in Supplementary Material). We expected that a durable memory phenotype would equate to decreased expression of transcripts associated with a fully differentiated T cell effector function profile. In most cases, as expected, both drug treatments downregulated effector transcripts including *Fos, JunB*, Granzyme B (*Gzmb*), and IFN-γ (*Ifng*), but interestingly the effector transcription factors *Tbx21, Eomes*, and *Nfatc4* increased with CAL-101 treatment (Figure 6A; Figure S3A in Supplementary Material).

We were intrigued by the high expression of stem memory genes *Lef1* and *Tcf7* in CAL-101-treated T cells, known to enhance their persistence and antitumor activity (6, 20, 31). We therefore followed up our RNA-seq data by assaying the protein levels of nuclear Lef1, Tcf7 and their upstream molecule β-catenin in CAL-101-treated T cells versus AKTi and vehicle T cells. Similar to our transcript expression results, nuclear β-catenin was similar between groups and both drug treatments had slightly higher, though not statistically significant Lef1. However, PI3Kδ blockade significantly increased nuclear Tcf7 over vehicle while AKTi did not (Figures 6B,C). Thus, our protein results confirm our findings with RNA-seq, and collectively suggest that while the phenotype of AKT inhibited T cells resembles that of potent central memory T cells, PI3Kδ blockade profoundly induced key stem memory transcripts and protein in human CAR T cells that might be fundamentally responsible for their longevity and potency *in vivo*.

In addition to memory transcripts that we hypothesized would be altered by CAL-101 treatment (designated as “Genes of Interest” see Figure S3A in Supplementary Material), we also detected other transcripts which were differentially expressed in CAL-101-primed T cells vs. vehicle (designated as “Discovered Gene Alterations” see Figure S3A in Supplementary Material). Many of these genes are associated with T cell fitness versus exhaustion including high antiapoptotic and metabolism transcripts *Bcl2, stradb* (or ILPIP), and *Ldlrap1* (Figure S3A in Supplementary Material). Interestingly, ILPIP is an antiapoptotic protein with energy-generating metabolism (33). Of additional interest, KLF4 was dramatically down regulated in T cell treated with CAL-101. This finding is important as this transcript has been previously reported to restrict memory CD8^+^ T cell responses to foreign antigen (34) (Figure S3A in Supplementary Material). Also, we found cell cycle proteins differentially regulated including cyclin D1 (*CCND1*) which was upregulated uniquely with CAL-101 treatment, and p21 (*CDKN1A*) which was downregulated compared to control especially by CAL-101 treatment. Since p21 prevents cyclin protein mediated progression from G1 to S (35) and we saw increased transcript of cyclin D1, suggesting that the increased cell frequencies of CAL-101 T cells after infusion may be due in part to less inhibited cell division (Figure S3A in Supplementary Material). Collectively based on our data, we suspect that CAL-101 induced Tcf7 signaling while concomitantly downregulating KLF4 may augment memory and antitumor efficacy by preventing the cells from undergoing terminal differentiation. Thus, CAL-101 regulates distinct pathways (such as Tcf7, KLF4, and ILPIP) potentially critical for supporting a less differentiated memory phenotype in adoptively transferred T cells.

## Discussion

**Figure 7:**
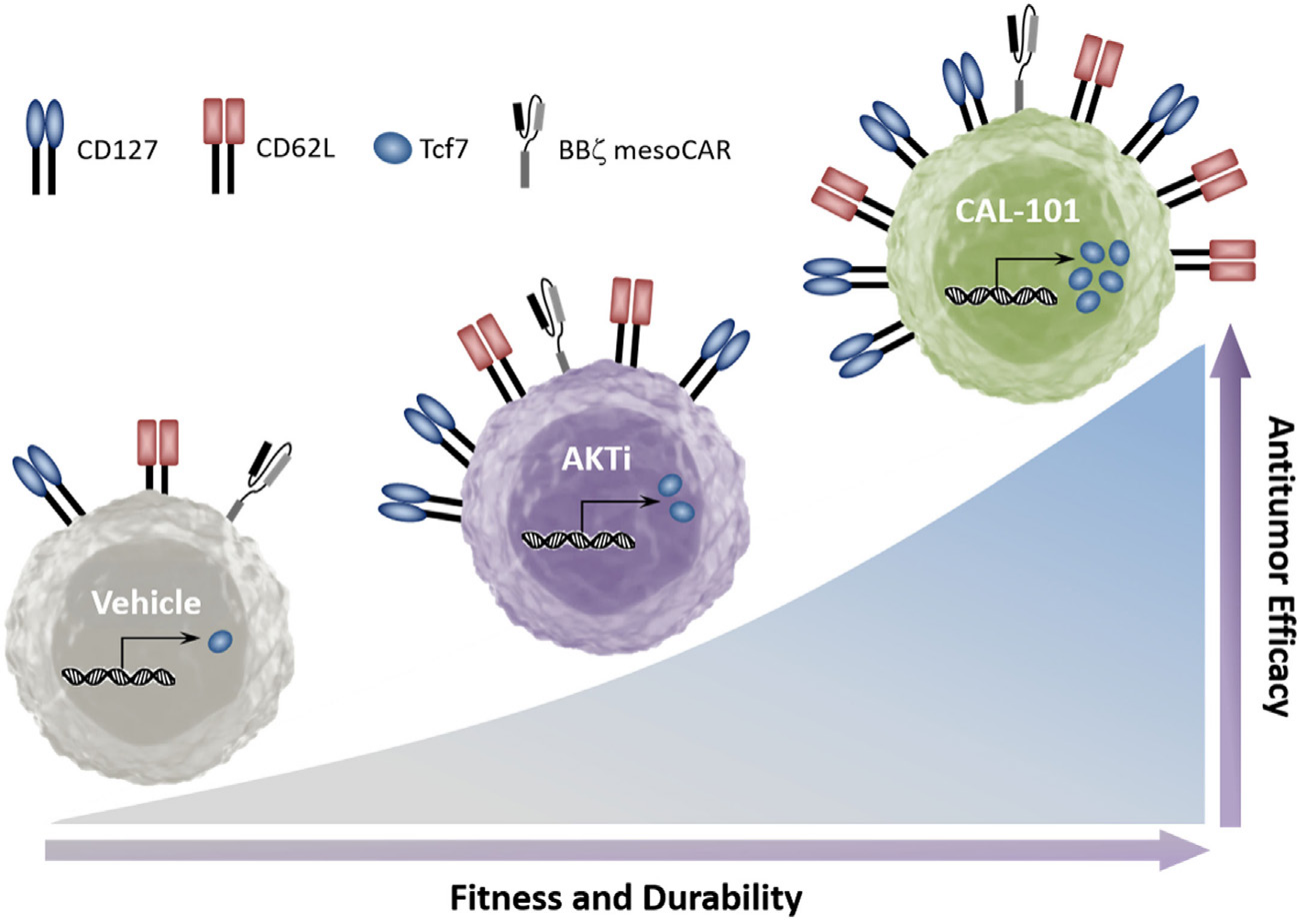
CAL-101 induces a strong memory phenotype and powerful antitumor capacity in chimeric antigen receptor (CAR) and TCR-reactive T cells. Graphical comparison of the memory capacity and antitumor efficacy of tumor-reactive T cells cultured in vehicle, AKT inhibitor (AKTi), or phosphatidylinositol-3-kinase p110δ (PI3Kδ) inhibitor (CAL-101).

Collectively, our data indicate that while PI3K is upstream of AKT, its blockade through inhibition of p110δ induces stronger antitumor capacity in infused CD8^+^ T cells than AKT inhibition. Furthermore, while inhibition of either PI3Kδ or AKT supports the generation of T cells with a central memory phenotype, we found that PI3Kδ blockade preferentially increases CD127 and the stem memory transcription factor Tcf7. Additionally, PI3Kδ blockade improved the antitumor response of both murine and human tumor-reactive T cells over that of traditionally expanded (vehicle) or AKTi-treated T cells (Figure 7).

Successful immunity against both viral infections and cancer requires long-term protection provided by memory cells (36). We have classically associated T cell memory with expression of surface markers such as CD62L, CCR7, and CD127 (28, 37), which were dramatically increased in CAL-101-treated T cells. We sought to determine whether CAL-101 induced CD62L and CD127 were responsible for the enhanced antitumor immunity after PI3Kδ blockade. We found early evidence to conclude that the longevity of immune responses exerted by CAL-101 T cells cannot be solely explained by high CD62L expression, as sorted CD62L^+^ T cells from vehicle expanded T cells cannot recapitulate the antitumor efficacy of either CAL-101-primed T cells or naive T cells enriched directly from the spleen. We suspect that uninhibited TCR and IL-2 signals drive vehicle CD62L^+^ cells to rapidly differentiate once transferred *in vivo* (38). While naive and CAL-101 T cells exerted similar antitumor immunity, it is important to note that the benefit of CAL-101 treatment is the preservation of a less differentiated phenotype while the T cells proliferate. Expanding tumor-reactive T cells to a high T cell yield for therapy is still vital to the therapy’s success (3, 31), and thus the low yield of unexpanded naive cells may be a disadvantage.

Additionally, *in vivo* blockade of IL-7 was insufficient to impair the antitumor efficacy of CAL-101-primed T cells. However, it is probable that the administration of the anti-IL-7 antibody did not completely deplete IL-7 and that the higher expression of CD127 on the CAL-101-primed T cells allowed for successful scavenging of this cytokine. Future studies using T cells from *Il7r* conditional knockout mice may determine the importance of CD127 to the longevity of CAL-101-primed T cell antitumor responses. In contrast, we found that IL-2 complex therapy was crucial to the efficacy of CAL-101 T cells to control tumor growth.

Since PI3Kδ blockade augmented antitumor potency in an apparent CD62L and IL-7 independent manner, we suspected that other aspects of T cell differentiation were being altered uniquely by CAL-101 treatment. One pathway consistently associated with durable memory T cells is the Wnt/β-catenin pathway, whether it will be active in stem memory T cells (6, 39) or in Th17 cells (40). We found that CAL-101 increased Tcf7 expression in human T cells significantly above vehicle while AKT inhibition did not. Thus, future studies with Tcf7 knockout T cells will help us understand the role and mechanism of Tcf7 in enhancing memory phenotype due to PI3Kδ blockade. Additionally, while our early genetic investigations indicate CAL-101-treated T cells have a unique phenotype compared to vehicle or AKTi-treated T cells, further genetic and epigenetic analysis will better elucidate whether this phenomenon is due to preservation of a naive-like phenotype, or true induction of a stem memory T cell phenotype.

Among the class 1A catalytic subunits of PI3K, P110δ is expressed mainly in the hematopoietic lineage (41) while P110α and β are thought to play a minimal role (42–44). Yet, while CAL-101 is highly specific for p110δ, we are cautious to attribute its potentiating effect on T cell memory phenotype solely to P110δ blockade, since CAL-101 also affects the class II, III, and IV PI3Kinases when used at doses similar to what we used to treat our T cells (45). Future experiments with P110δ knockout mice with or without CAL-101 treatment will further elucidate the way in which CAL-101 primes T cells to exert more powerful antitumor immunity.

Inducing a durable memory phenotype through reversible pharmaceutical manipulation of PI3Kδ is an attractive way of generating potent T cells. Yet, while CAL-101 T cells exerted a longer antitumor response than vehicle and AKTi-treated T cells, they eventually lost control of the tumor in most mice (Figure 2B). We found that as early as one week after transfer, these cells were expressing similar levels of PD-1 and KLRG-1 compared to vehicle and AKTi-treated T cells (Figure 2A). Thus, while PI3Kδ blockade induces a strong memory phenotype including high Tcf7 expression, it does not appear to protect the cells from inhibitory signaling and exhaustion. We posit that a combination of PD-1 checkpoint blockade therapy may prevent the reestablishment of immune tolerance and subsequent tumor progression observed following treatment with CAL-101 T cells allowing for continued tumor control. Additionally, since CAL-101’s direct cytotoxic effects on cancers (46) as well as its ability to break immune tolerance without affecting effector T cell viability (19, 44) are well known, systemic administration of CAL-101 may synergize well with current checkpoint blockade or vaccine strategies. Nonetheless, whatever the route of application may be, we propose the use of CAL-101 as a viable and potent method for enhancing immune-based therapies for cancer to improve patient responses and survival.

## Ethics Statement

Studies were approved by the IACUC of the Medical University of South Carolina Animal Resource Center (ARC #3039). Deidentified human PBMCs and tumor samples were collected under approval of the MUSC Internal Review Board (pro13570). Human T cells were engineered *via* approval from the Institutional Biosafety Committee (#2335).

## Author Contributions

Conceptualization and methodology: JB, KM, and CP. Investigation: JB, MN, MW, and AS. Formal analysis: JB, BA, and JH. Writing original draft: JB and CP. Writing, reviewing, and editing: JB, KM, MN, BA, MW, AS, SB, LN, JH, and CP. Visualization: JB, SB, and CP. Funding acquisition and resources: JB, MN, SB, and CP. Supervision and project administration: CP.

## Conflict of Interest Statement

The authors declare that the research was conducted in the absence of any commercial or financial relationships that could be construed as a potential conflict of interest.

## Supplementary Material

Figure S1 Depletion of IL-7 does not alter numbers or memory phenotype of donor CAL-101 T cells. **(A)** Individual tumor burden (mm^2^) and **(B)** percent survival of mice which received no T cell treatment, or 8 × 10^5^ pmel-1 CD8^+^ T cells primed with vehicle, 1 or 10 µM AKTi or CAL-101; *n* = 10–12 mice/group. Kaplan–Meier curve analyzed by log rank test; ns, not statistically significant, **p* < 0.05, *****p* < 0.0001. **(C)** Growth of human peripheral T cells primed with vehicle (DMSO), 1 or 10 µM AKTi, and 1 or 10 µM CAL-101 8 days following antigen stimulation; *n* = 3 independent cultures. One-way repeated measures ANOVA; **p* < 0.05.

Figure S2 Depletion of IL-7 does not alter numbers or memory phenotype of donor CAL-101 T cells. **(A)** Analysis of *in vivo* numbers of transferred pmel-1 T cells primed with vehicle, AKTi, or CAL-101 10 days after transfer into mice receiving isotype or IL-7 depleting antibody, *n* = 5 mice/group from one experiment. One way repeated measures ANOVA; ns, not statistically significant, ***p* < 0.01, ****p* < 0.001. **(B,C)** Memory phenotype of transferred pmel-1 T cells primed with vehicle, AKTi, or CAL-101 10 days after transfer into **(B)** isotype or **(C)** IL-7-depleted mice; *n* = 5 mice/group from one experiment. One-way repeated measures ANOVA; ns, not statistically significant, **p* < 0.05, ***p* < 0.01, *****p* < 0.0001.

Figure S3 Differential expression of genes in CAL-101- or AKTi-treated T cells. **(A)** Differential expression of genes hypothesized to be altered in expression (Genes of Interest) as well as genes discovered to be markedly altered (Discovered Gene Alterations) in CAL-101- or AKTi-treated cells compared to vehicle using RNA sequencing; *n* = 3 normal donors.
