# Supplementary figures and images for: PI3Kδ Inhibition Enhances the Antitumor Fitness of Adoptively Transferred CD8+ T Cells
Source: Front Immunol. 2017 Sep 29;8:1221. doi: 10.3389/fimmu.2017.01221 (PMC5626814; doi:10.3389/fimmu.2017.01221)

**
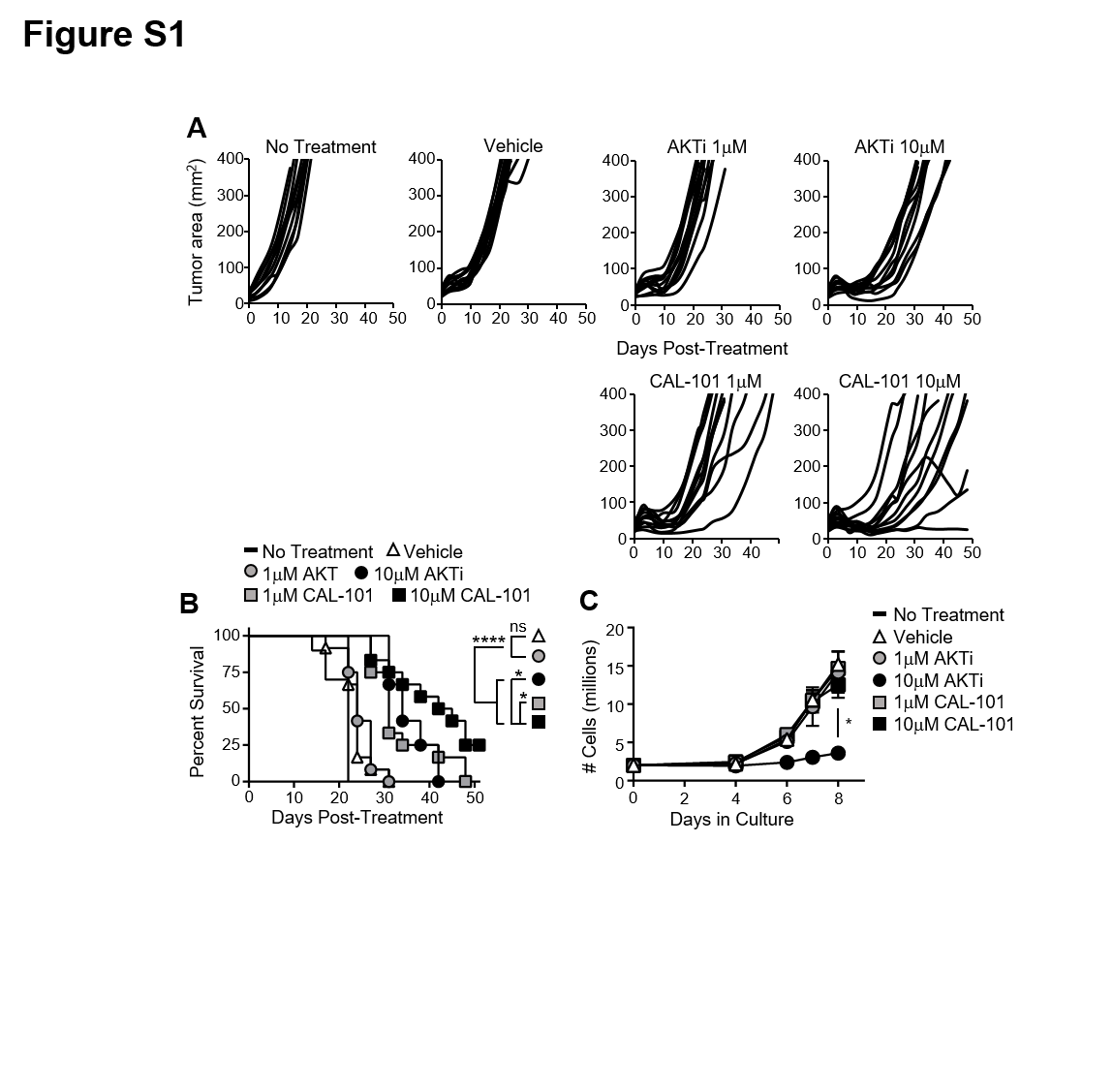
**

**
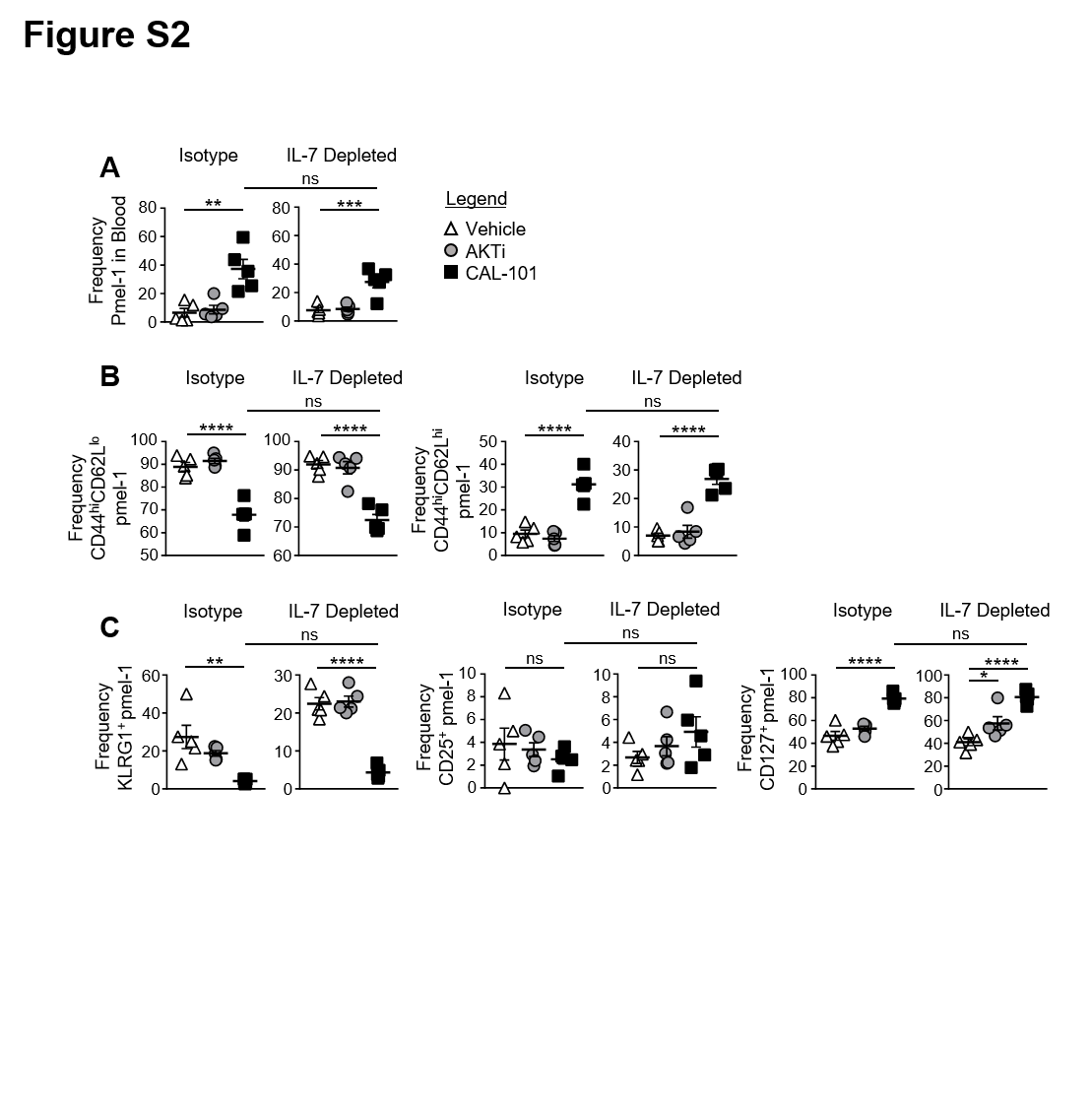
**


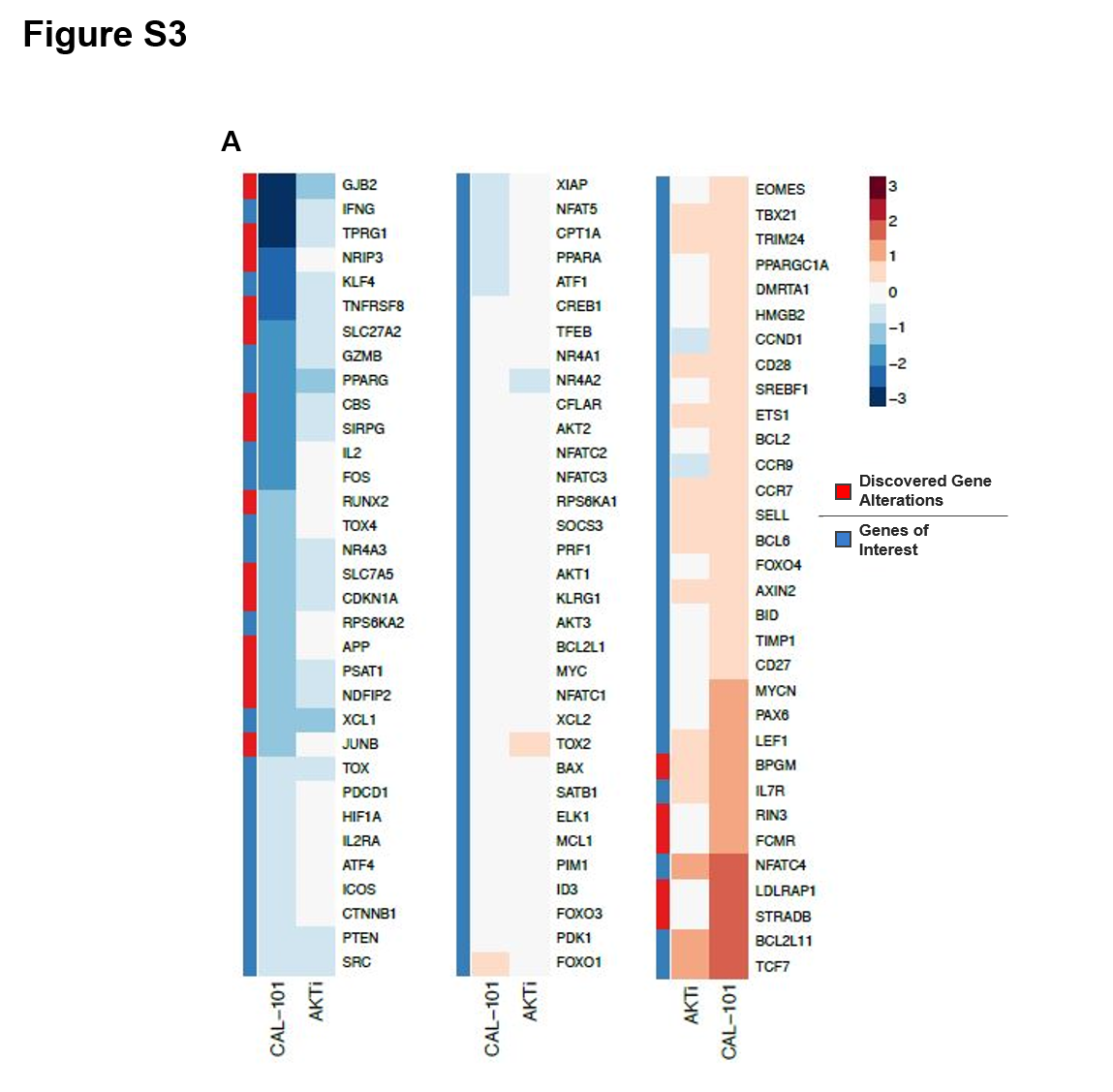

Supplement: Figure S1 — Depletion of IL-7 does not alter numbers or memory phenotype of donor CAL-101 T cells. (A) Individual tumor burden (mm2) and (B) percent survival of mice which received no T cell treatment, or 8 × 10^5^ pmel-1 CD8+ T cells primed with vehicle, 1 or 10 µM AKTi or CAL-101; n = 10–12 mice/group. Kaplan–Meier curve analyzed by log rank test; ns, not statistically significant, *p < 0.05, ****p < 0.0001. (C) Growth of human peripheral T cells primed with vehicle (DMSO), 1 or 10 µM AKTi, and 1 or 10 µM CAL-101 8 days following antigen stimulation; n = 3 independent cultures. One-way repeated measures ANOVA; *p < 0.05. [file Data_Sheet_1.DOCX]
